# Comparison between continuous and discrete doses for model based designs in cancer dose finding

**DOI:** 10.1371/journal.pone.0210139

**Published:** 2019-01-09

**Authors:** Márcio Augusto Diniz, Mourad Tighiouart, André Rogatko

**Affiliations:** Biostatistics and Bionformatics Research Center, Samuel Oschin Comprehensive Cancer Institute, Los Angeles, California, United States of America; University Medical Center Gottingen, GERMANY

## Abstract

Despite of an extensive statistical literature showing that discretizing continuous variables results in substantial loss of information, categorization of continuous variables has been a common practice in clinical research and in cancer dose finding (phase I) clinical trials. The objective of this study is to quantify the loss of information incurred by using a discrete set of doses to estimate the maximum tolerated dose (MTD) in phase I trials, instead of a continuous dose support. Escalation With Overdose Control and Continuous Reassessment Method were used because they are model-based designs where dose can be specified either as continuous or as a set of discrete levels. Five equally spaced sets of doses with different interval lengths and three sample sizes with sixteen scenarios were evaluated to compare the operating characteristics between continuous and discrete dose designs by Monte Carlo simulation. Loss of information was quantified by safety and efficiency measures. We conclude that if there is insufficient knowledge about the true MTD value, as commonly happens in phase I clinical trials, a continuous dose scheme minimizes information loss. If one is required to implement a design using discrete doses, then a scheme with 9 to 11 doses may yield similar results to the continuous dose scheme.

## Introduction

Measurements of continuous variables are made in all fields of medicine. In medical research such continuous variables are often converted into categorical variables by grouping values into two or more categories in order to have easier interpretations. Cox [1] presented the first optimization criterion for discretizing a continuous variable showing the minimum loss of information as a function of the number of categories. Since then, several authors [2–7] have pursued methodologies to provide optimal criteria of discretization for continuous variables based on test statistics. On the other hand, extensive statistical literature [8–14] has advised against categorization due the loss of power and precision of the estimated quantities.

This debate has been ignored for cancer phase I clinical trials. Phase I trials are the first step of translation of a new drug from laboratory research to clinical practice. The aim in phase I trials is to identify a maximum tolerable dose (MTD) for subsequent phase II and III trials. The compromise underlying the design of cancer phase I clinical trials is that reaching the MTD as fast as possible while at the same time avoiding unacceptable toxic events. [15] Traditionally, dose finding has been designed according to the 3 + 3 principle and its variants, which was first described by Dixon and Mood [16] and requires a pre-specified set of discrete doses. Following the up-and-down approach, a large collection of methods estimates the MTD of a new agent using a pre-specified set of doses. However, Hu et al. [17] and Chu et al. [18] pointed out that the common assumption underlying almost all existing methods is that one of the pre-specified doses is the MTD does not hold in practice because often only limited prior information regarding the dose-toxicity relationship of the experimental drug is available prior to the first-in-human trial.

Although the use of rule-based designs still prevails, model-based designs such as the continual reassessment method (CRM) introduced by O’Quigley et al. [19] and Escalation With Overdose Control (EWOC) by Babb et al. [20] have been gaining popularity in clinical practice. [21] In these model-based designs, a parametric model is used to describe the relationship between the probability of a dose-limiting toxicity (DLT) and the dose level of the new agent, which is either a continuous or a discrete variable. Even though intravenous drugs are still more prevalent than oral drugs [22, 23], clinical trials using continuous dose [24, 25] are less often performed since clinicians are used to the up-and-down approach.

In this work, a Monte Carlo simulation study to compare the operating characteristics of continuous and discrete dose using model-based designs is presented. The loss of information is evaluated using the statistical measures bias and mean squared error as well as specific measures for phase I clinical trials to quantify safety and efficacy of the trial. Even though, results are presented for EWOC and CRM, our goal is not to compare these two methods. Other authors (e.g., Chu et al. [26]) have compared the performance of different versions of CRM and EWOC. This article is organized as follows. In the next section, the EWOC design is briefly introduced. Then, a simulation study is described and its results are presented with discussion.

## CRM and EWOC

Let *X*_*min*_ and *X*_*max*_ denote the minimum and maximum dose levels available for use in the trial. Note that the dose given to the first cohort of patients is not necessarily equal to *X*_*min*_ but there must be strong evidence that it is a safe dose.

In this way, the minimum and maximum doses are the lower and upper bound of the support of the MTD *γ*, which is defined by

$$\begin{array}{c} P(DLT|dose=\gamma )=\theta , \end{array}$$ (1)

such that *θ* is defined as the target toxicity level corresponding to the expected proportion of patients to experience a medically unacceptable, dose-limiting toxicity if the MTD *γ* is administered. The relationship between toxicity and dose level is defined as

$$\begin{array}{c} P\left(DLT|dose=x\right)=F\left(\beta_{0}+\beta_{1}x\right), \end{array}$$ (2)

where *F* is a specified distribution function, and *β*_0_, *β*_1_ are unknown parameters such that *β*_1_ > 0. The usual choice is the Logistic distribution, *F*(*u*) = (1 + *exp*(−*u*))^−1^. Following (1) and (2), the MTD will be given by

$$\begin{array}{ccc} \gamma & = & \frac{F^{-1}\left(\theta \right)-\beta_{0}}{\beta_{1}}\\ & = & X_{min}+\frac{F^{-1}\left(\theta \right)-F^{-1}\left(\rho_{0}\right)}{\beta_{1}}, \end{array}$$ (3)

where *ρ*_0_ denotes the probability of a DLT at the minimum dose. Using the definition of the MTD and probability of toxicity at initial dose, one can show that

$$\begin{array}{ccc} \beta_{0} & = & \frac{\gamma F^{-1}\left(\rho_{0}\right)-X_{min}F^{-1}\left(\theta \right)}{\gamma -X_{min}},\\ \beta_{1} & = & \frac{F^{-1}\left(\theta \right)-F^{-1}\left(\rho_{0}\right)}{\gamma -X_{min}}. \end{array}$$ (4)

Denote by *y*_*i*_ the toxicity response (1 for DLT and 0 for no DLT) of the *ith* patient. The likelihood of the data *D*_*k*_ = {(*x*_*i*_, *y*_*i*_), *i* = 1, …, *k*} after observation of *k* patients is

$$\begin{array}{c} L\left(\rho_{0},\gamma |D_{k}\right)=\prod_{i=1}^{k}F{\left(\beta_{0}+\beta_{1}x_{i}\right)}^{y_{i}}{\left[1-F\left(\beta_{0}+\beta_{1}x_{i}\right)\right]}^{1-y_{i}}. \end{array}$$ (5)

for (*β*_0_, *β*_1_) defined as funtions of (*ρ*_0_, *γ*) given in (4).

Prior information is incorporated for (*ρ*_0_, *γ*) under the restrictions of *γ* ∈ [*X*_*min*_, *X*_*max*_] and *ρ*_0_ ∈ (0, 1). Since the parameter space is bounded, a flexible choice is a *Beta*(*a*_*ρ*_, *b*_*ρ*_) distribution for *ρ*_0_ and a re-scaled *Beta*(*a*_*γ*_, *b*_*γ*_) distribution for *γ* can represent prior beliefs. Tighouart et al. [27] examined a large class of prior distributions that also could be considered. Uniform distributions will be used for both parameters for simplicity.

Finally, the calculation of the posterior distribution for (*ρ*_0_, *γ*) can be evaluated [28] and implemented using numerical integration and a Markov chain Monte Carlo sampler

$$\begin{array}{c} \pi \left(\rho_{0},\gamma |D_{k}\right)=c\left(D_{k}\right)L\left(\rho_{0},\gamma |D_{k}\right)\pi \left(\rho_{0},\gamma \right), \end{array}$$ (6)

where *c*(*D*_*k*_) is a normalizing constant. The choice of the next dose based on the posterior information depends on the design.

### CRM

Following [19], the (*k* + 1)st patient receives the dose given by

$$\begin{array}{c} \underset{x_{k+1}}{\text{arg}\;\text{min}}\left|\theta -F\left(x_{k+1}\hat{\beta }\right)\right| \end{array}$$ (7)

where $\hat{\beta }$ is the posterior estimate obtained from *γ* posterior distribution using (4).

For a discrete set of doses, both designs are performed using continuous dose with an additional step that one could either round down *x*_*k*+1_ to the closest dose prioritizing safety or round to the nearest dose preferring the ability to explore the available set of doses. Notice that there are many versions of CRM (i.e., proposed by [29–31]) with different models and algorithms, but they follow the same principle of assigning the next patient a dose that has an an estimated toxicity probability closest to the target toxicity level *θ*.

### EWOC

Following Babb et al. [20], the (*k* + 1)st patient receives the dose given by the *α*-quantile of the *γ* posterior distribution

$$\begin{array}{c} x_{k}=\Pi^{-1}\left(\alpha |D_{k}\right), \end{array}$$ (8)

where *α* is the probability that the dose selected by EWOC is higher than the MTD.

The feasibility bound could vary during the trial as discussed by Tighiouart and Rogatko [32]. The rationale behind this approach is that uncertainty about the MTD is high at the onset of the trial and a small value of *α* offers protection against the possibility of administering dose levels much greater than the MTD. As the trial progresses, uncertainty about the MTD declines and the likelihood of selecting a dose level significantly above the MTD become significantly smaller.

There are several suggestions for the choice of the feasibility boundary. Originally Babb et al. [20] suggested a fixed feasibility boundary *α* equal to 0.25. Babb and Rogatko [33] suggested an increasing feasibility boundary until 0.5 with initial *α* equal 0.25. Wheeler et al. [34] suggested a similar strategy, but conditional on the previous patient having no DLT, denoted by C(0.25, 0.05).

## Simulation study

Both designs were applied for continuous dose and discrete dose. The minimum and maximum doses were standardized as *X*_*min*_ = 0 and *X*_*max*_ = 1. Considering the discrete dose, five equally spaced sets with different interval lengths between two doses given by 0.05, 0.10, 0.2 and 0.25 were established: D0.05 = {0, 0.05, …, 0.95, 1} has 21 doses, D0.10 = {0, 0.10, …, 0.90, 1} has 11 doses, D0.125 = {0, 0.125, …, 0.875, 1} has 9 doses, D0.20 = {0, 0.20, …, 0.80, 1} has 6 doses and D0.25 = {0, 0.25, 0.50, 0.75, 1} has 5 doses. The working model is the logistic model. The feasibility strategy C(0.05, 0.05) was applied. There are 12 (4 true MTD × 3 sample sizes) simulations for each dose scheme and true model.

**Fig 1:**
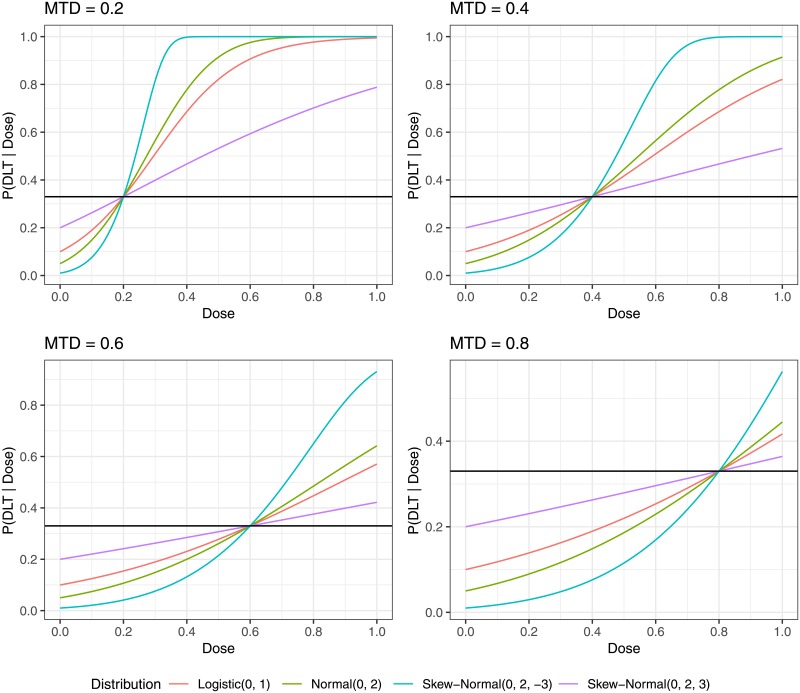
Probability of DLT as function of dose.

The target toxicity level *θ* was set equal to 0.33, with four true values for the MTD = {0.2, 0.4, 0.6, 0.8} and four true distributions Logistic(*μ* = 0, *σ*^2^ = 1), Normal(*μ* = 0, *σ*^2^ = 2), Skew-Normal($\mu =0,\sigma^{2}=\sqrt{2},\lambda =3$) and Skew-Normal($\mu =0,\sigma^{2}=\sqrt{2},\lambda =-3$), where the parameters *μ*, *σ*^2^, λ are mean, variance and skewness, respectively. The distributions are illustrated in Fig 1.

Three different sample sizes *n* = 20, 40, 60 with cohorts of one patient were treated. In the discrete dose scheme, the rounding to the nearest dose approach was applied and skipping doses was not allowed.

A Monte Carlo study was performed with 1000 replicates for each study design. Posterior distribution was sampled using JAGS [35], in particular the algorithm Slice Sampling, with 5000 iterations for the adapt phase, and 10000 iterations for burn-in resulting in a sample of 1000 values for each parameter of interest without thinning.

Bias and the root of mean square error (RMSE) of the MTD estimate were calculated to compare accuracy and precision across scenarios. Average DLT rate and percentage of trials in which DLT proportion is inside the target toxicity level interval defined as *θ* ± 0.05 were calculated to quantify safety. In addition, the correct MTD recommendation was quantified using the percentage of trials with the estimated MTD within the optimal MTD interval defined as

$$\begin{array}{c} \{\text{dose}:\gamma -0.10\times \gamma <\text{dose}<\gamma +0.10\times \gamma \}, \end{array}$$ (9)

and the optimal target toxicity level interval defined as

$$\begin{array}{c} \{\text{dose}:\theta -0.10<F(\text{dose})<\theta +0.10\}, \end{array}$$ (10)

and the percentage of patients receiving optimal doses defined by those optimal intervals.

Notice that these two criteria of optimality for doses are different measures of the distance between the true MTD and a dose. The optimal MTD interval defines a interval around the MTD, while the optimal toxicity interval defines a interval around the target toxicity level. From the perspective of a patient participating in a dose finding trial, the best design is the one with the highest proportion of patients receiving optimal doses. Therefore, it is important to characterize the discrete dose schemes based on the number of possibles doses that could be considered optimal.

Table 1 presents the number of possible optimal doses using the optimal MTD interval for all four true distributions. Dose schemes D0.05 and D0.10 for some true MTD values present more than one dose which is an optimal dose. The dose scheme D0.25 does not contain any possible dose which could be considered as an optimal dose for the true MTD values 0.2, 0.4 and 0.6. The dose scheme D0.125 does not contain any possible dose that is considered optimal dose for the true MTD value 0.2, but it contains more than one dose that can be considered optimal dose for the true MTD value 0.8.

**Table 1:** Percentage (Number) of doses relative to each discrete dose scheme which are optimal doses based on the optimal MTD interval (True MTD ± 0.15 × True MTD).

True MTD	Optimal Interval	Dose scheme				
		D0.05	D0.10	D0.125	D0.20	D0.25
0.2	(0.17; 0.23)	4.8 (1)	9.1 (1)	0.0 (0)	16.7 (1)	0.0 (0)
0.4	(0.34; 0.46)	14.3 (3)	9.1 (1)	11.1 (1)	16.7 (1)	0.0 (1)
0.6	(0.51; 0.69)	14.3 (3)	9.1 (1)	11.1 (1)	16.7 (1)	0.0 (0)
0.8	(0.68; 0.92)	23.8 (5)	27.3 (3)	22.2 (2)	16.7 (1)	20.0 (1)

The dose schemes are evaluated based on the optimal toxicity interval in Table 2. All discrete dose schemes contain at least one optimal dose under the definition of the optimal toxicity interval if the true distribution is logistic(0, 1) or skew-normal(0, 2, 3). There are scenarios where the dose schemes D0.125 and D0.25 do not contain any optimal toxicity dose for the normal(0, 2), and skew-normal(0, 2, -3) distributions. If the toxicity optimal interval is defined as *θ* ± 0.05 instead of *θ* ± 0.10, then the results for optimal toxicity interval for logistic distribution are identical to the results in Table 1.

**Table 2:** Percentage (Number) of doses relative to each discrete dose scheme which are optimal doses based on the optimal toxicity interval (*θ* ± 0.10).

True Distribution	True MTD	Dose scheme				
		D0.05	D0.10	D0.125	D0.20	D0.25
logistic(0, 1)	0.2	14.3 (3)	9.1 (1)	11.1 (1)	16.7 (1)	20.0 (1)
	0.4	23.8 (5)	27.3 (3)	22.2 (2)	16.7 (1)	20.0 (1)
	0.6	38.1 (8)	36.4 (4)	33.3 (3)	33.3 (2)	40.0 (2)
	0.8	47.6 (10)	45.5 (5)	44.4 (4)	50.0 (3)	40.0 (2)
normal(0, 2)	0.2	4.8 (1)	9.1 (1)	0.0 (0)	16.7 (1)	0.0 (0)
	0.4	14.3 (3)	9.1 (1)	11.1 (1)	16.7 (1)	0.0 (0)
	0.6	23.8 (5)	27.3 (3)	22.1 (2)	16.7 (1)	20.0 (1)
	0.8	33.3 (7)	27.3 (3)	33.3 (3)	16.7 (1)	20.0 (1)
skew-normal(0, 2, -3)	0.2	4.8 (1)	9.1 (1)	0.0 (0)	16.7 (1)	0.0 (0)
	0.4	9.5 (2)	9.1 (1)	11.1 (1)	16.7 (1)	0.0 (0)
	0.6	14.3 (3)	9.1 (1)	11.1 (1)	16.7 (1)	0.0 (0)
	0.8	19.0 (4)	18.2 (2)	22.2 (2)	16.7 (1)	20.0 (1)
skew-normal(0, 2, 3)	0.2	23.8 (5)	27.3 (3)	22.2 (2)	16.7 (1)	20.0 (1)
	0.4	42.9 (9)	45.5 (5)	33.3 (3)	50.0 (3)	20.0 (2)
	0.6	61.9 (13)	63.6 (7)	55.6 (5)	50.0 (3)	40.0 (2)
	0.8	61.9 (13)	63.6 (7)	55.6 (5)	66.7 (4)	60.0 (3)

All the simulations were performed using the R-package EWOC with the development branch CRM available at GitHub [36].

## Results

**Fig 2:**
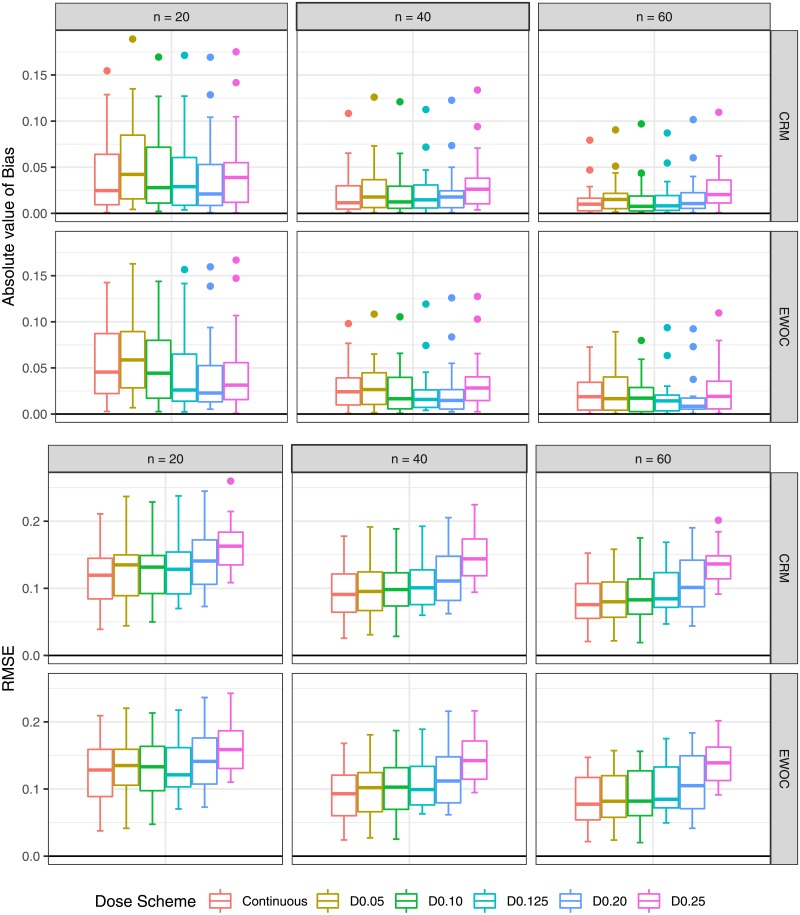
Absolute bias and RMSE as a function of sample size and dose scheme.

**Fig 3:**
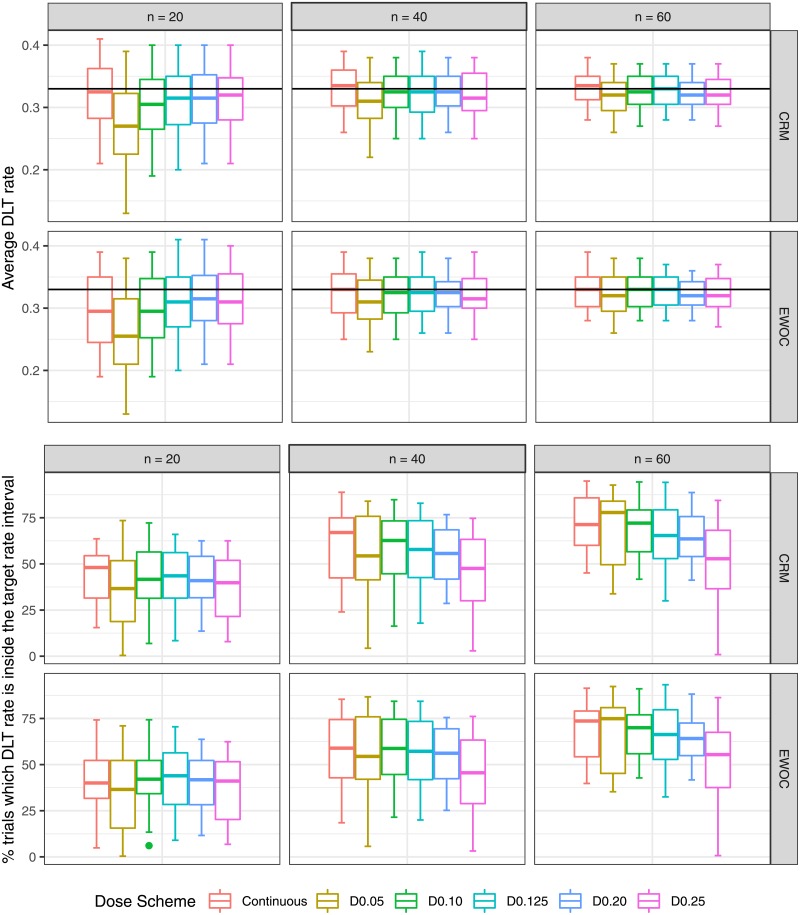
DLT average and percentage of trials such that the observed DLT probability is inside the interval [*θ* − 0.1; *θ* + 0.1]as a function of true distribution and dose scheme.

**Fig 4:**
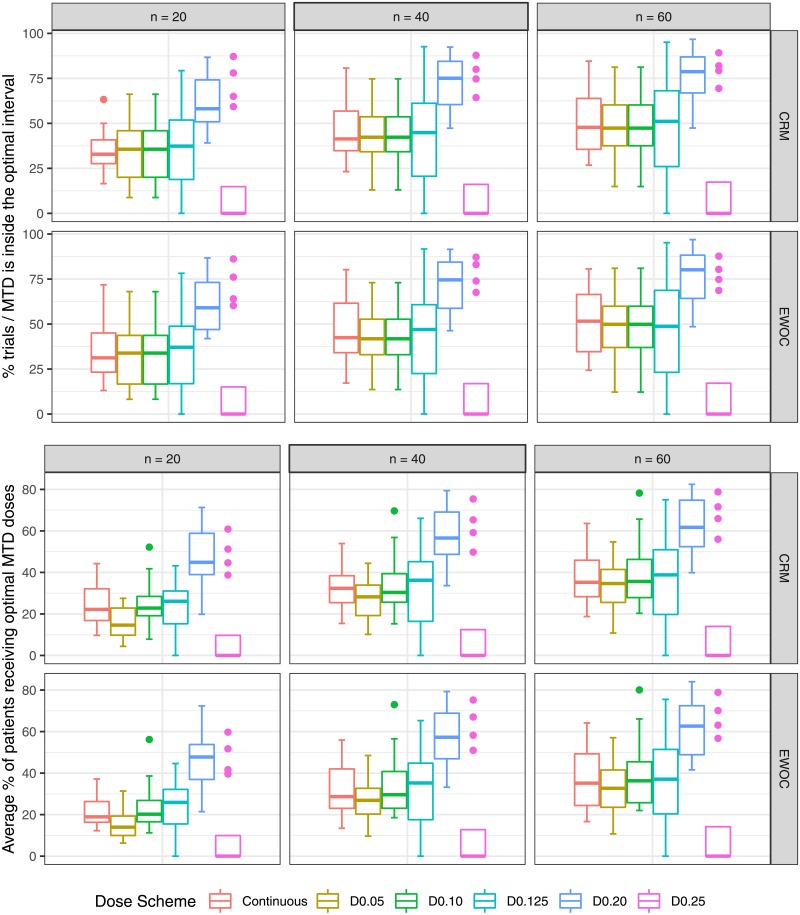
Percentage of trials with the estimated MTD inside the optimal MTD interval and average percentage of patients receiving doses inside the optimal MTD interval as a function of true sample size and dose scheme.

**Fig 5:**
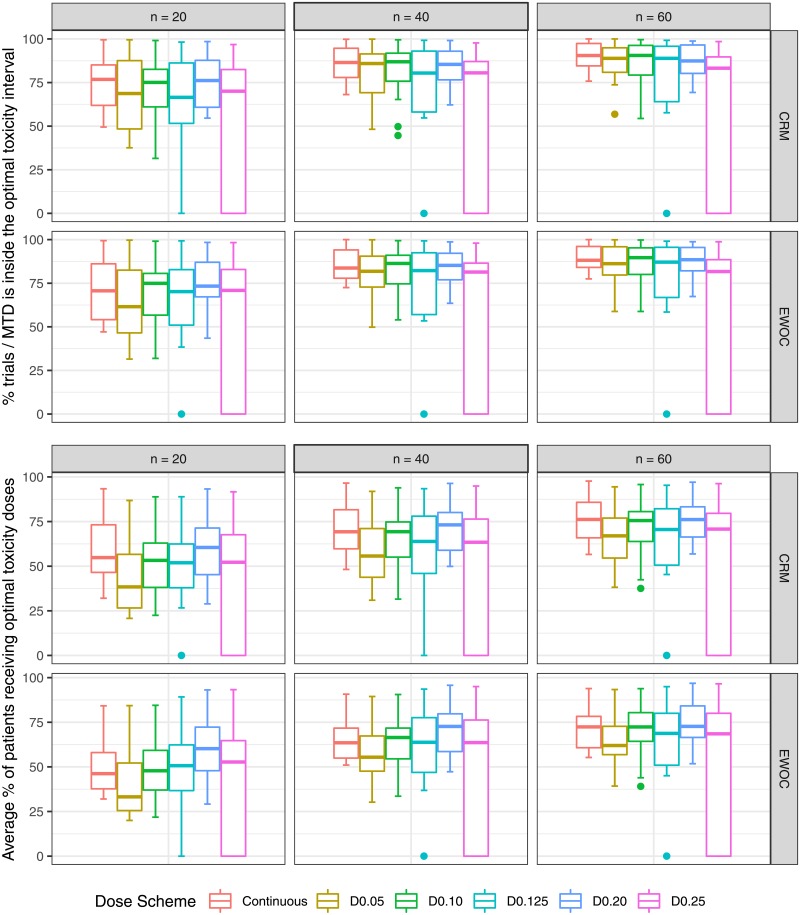
Percentage of trials with the estimated MTD inside the toxicity optimal interval and average percentage of patients receiving doses inside the optimal toxicity interval as a function of true distribution and dose scheme.

The results are evaluated based on median and quantiles over 16 scenarios (4 True MTD × 4 True Distributions). Absolute bias and RMSE are in Fig 2. Average DLT rate and percentage of trials in which the DLT proportion is inside the target toxicity level are in Fig 3. Percentage of trials in which the estimated MTD is inside the optimal MTD interval and average percentage of patients receiving doses inside the optimal MTD interval are in Fig 4. Percentage of trials in which the estimated MTD is inside the optimal toxicity interval and average percentage of patients receiving doses inside the optimal toxicity interval are in Fig 5.

### Bias

The differences in absolute bias are negligible among the dose schemes for both designs. It is still possible to observe some patterns. **CRM:** For *n* = 20, continuous dose presents a lower median value of absolute bias than D0.05, D0.25, and a close median value to D0.10, D0.125, D0.20. The same pattern is observed when *n* = 40, 60, with D0.25 showing the highest values. **EWOC:** For *n* = 20, the continuous dose presents a lower median value of absolute bias than D0.05, a close value to D0.10, and a higher value than D0.25. If *n* = 40, continuous dose, D0.05 and D0.25 show comparable median values of absolute bias, and D0.10 has lower values than all three. When *n* = 60, continuous dose, D0.05, D0.10, and D0.25 have similar median values to each other. The dose schemes D0.125 and D0.20 show the lowest median values of absolute bias for all sample sizes.

### RMSE

**CRM:** The median values of RMSE of all discrete designs are higher than the continuous dose for all sample sizes. The dose schemes D0.05, D0.10 and D0.125 show median values of RMSE that approximate to the continuous dose as the sample size increases. In addition, D0.20 and D0.25 have the poorest performance for all sample sizes, with D0.25 displaying the highest values. **EWOC:** The dose schemes D0.05, D0.10 and D0.125 display median values of RMSE that are slightly higher than the continuous dose for all sample sizes, except D0.125 when *n* = 20. In addition, D0.20 and D0.25 have poorer performance than for the continuous dose as the sample size increases.

### Average DLT rate

**CRM:** When *n* = 20, the continuous dose presents a median value closer to the target toxicity level compared to all discrete dose schemes, with D0.05 displaying the lowest value. For *n* = 40, the median value for the continuous dose exceeds the target toxicity level *θ*; D0.10, D0.125, D0.20 present median values close to *θ*; and D0.05, D0.25 have the lowest median values. For *n* = 60, the continuous dose still exceeds the target toxicity level; the median value of D0.125 reaches *θ* followed closely by D0.10, D0.05, D0.125 and D0.25. **EWOC:** All dose schemes present median values below the target toxicity level *θ* when *n* = 20, with D0.05 showing lower values than all other dose schemes. For *n* = 40, the median value of the continuous dose reaches the target toxicity level followed by D0.10, D0.125, and D0.20. When *n* = 60, the dose schemes D0.10 and D0.125 also reach the target toxicity level, in addition to the continuous dose. Moreover, D0.05, D0.20 and D0.25 show slightly lower median values than *θ*.

### Percentage of trials in which DLT proportion is inside the target toxicity level interval

**CRM:** The continuous dose shows higher median value than all discrete dose schemes when *n* = 20, 40. When *n* = 60, D0.05 outperforms the continuous dose; D0.10 is similar to the continuous dose, followed by D0.125, D0.20, and D0.25 dose schemes. **EWOC:** Similar median percentages are found to all dose schemes when *n* = 20. However, the performance of discrete designs decreases compared to the continuous dose as the sample sizes increases, except D0.05 which displays similar performance to the continuous dose for all sample sizes. D0.25 shows notably worse performance than all discrete dose schemes when *n* = 40, 60.

### Percentage of trials in which the estimated MTD is inside the optimal MTD interval

**CRM and EWOC:** The continuous dose presents median percentages close to D0.05, D0.10, D0.125 when *n* = 20, 40, 60. The dose scheme D0.20 surpasses all the other dose schemes, and D0.25 shows the poorest performance for all sample sizes. It is noteworthy that D0.125 presents values equal to zero for 50% of the scenarios as expected based on Table 1.

### Average percentage of patients receiving optimal MTDs

**CRM and EWOC:** The continuous dose presents a median percentage close to D0.10, higher than D0.05, and lower than D0.125 for *n* = 20; when *n* = 40, D0.05 approximates to the continuous dose; when *n* = 60, D0.125 also approaches the continuous dose. On the other hand, D0.20 surpasses all the dose schemes for all sample sizes. As expected based on Table 1, D0.25 underperforms all other designs and D0.125 shows 50% of scenarios with values equal to zero.

### Percentage of trials with the estimated MTD inside the toxicity optimal interval

**CRM:** When *n* = 20, the median percentage of the continuous dose is similar to D0.10, D0.20 and higher than D0.05, D0.125 and D0.25; D0.05 and D0.125 approach the continuous dose when *n* = 40, 60 respectively. Furthermore, D0.125 show values equal to zero for a few scenarios. The variability of performance for D0.25 is large and the median value lower than the continuous dose for *n* = 40, 60, with some values equal to zero. **EWOC:** All dose schemes present similar median values to each other, except D0.05 when *n* = 20 and D0.25 when *n* = 60. The results under D0.25 and D0.125 are more variable than those of other dose sets.

### Average percentage of patients receiving toxicity optimal interval

**CRM:** The continuous dose presents a median value close to D0.10, D0.125 and D0.25, higher than D0.05, and lower than D0.20 for *n* = 20; the continuous dose, D0.10, D0.20 are similar to each other, and higher than D0.05, D0.125 and D0.25 for *n* = 40, 60. **EWOC:** The continuous dose presents median value close to D0.10, D0.125 and D0.25, higher than D0.05, and lower than D0.20 for *n* = 20, 40. When *n* = 60, the continuous dose, D0.10, D0.125 and D0.25 approximate to D0.20. For both designs, D0.25 and D0.125 show a large variability in performance, with values equal to zero as expected based on Table 2. Moreover, D0.05 underperforms other dose schemes for all sample sizes.

## Discussion

EWOC and CRM are model-based designs where dose can be specified either as continuous or as a set of discrete levels. Therefore, they offer an ideal framework to compare the loss of information incurred by using a discrete set of doses to estimate the MTD in phase I trials, instead of a continuous dose support. This work compared such dose schemes considering sixteen scenarios and three samples sizes, with a conditional feasibility strategy for EWOC. The six doses schemes were one with continuous dose and five different pre-specified set of doses. The set of doses are equally spaced between the minimum and maximum doses, with two dose schemes that do not contain the true MTD values. The dose schemes were evaluated based on safety and efficiency measures.

Phase I clinical trials usually are performed with 5 or 6 doses chosen based on arbitrary criteria. Based on the simulations, discrete dose schemes containing 9 or 11 doses equally spaced between the minimum and maximum doses produce operating characteristics similar to the continuous dose. A dose scheme containing 6 pre-specified doses performed well, but with an increased RMSE. Nonetheless, the assumption that the pre-specified set of doses contains the true MTD is essential to obtain acceptable operating characteristics such as: the percentage of trials such that the MTD is inside the optimal MTD and toxicity intervals, and average percentage of patients receiving optimal MTD and toxicity doses. The challenge with this assumption is that it cannot be verified in the real world, outside a simulation setup.

Theoretically, the probability of selecting a point for a continuous random variable is equal to zero. Thus, defining a pre-specified set of doses that contains the exact true MTD value seems improbable. Notwithstanding, increasing the number of doses could be adopted as a possible solution to increase the chance that the pre-specified set of doses is close to the true MTD. On the other hand, a large number of doses can generate inefficient designs with low operating characteristics as was observed for the dose scheme D0.05.

Discrete dose schemes with 9 to 11 doses produce fine enough grids of the dose support to present similar results to the continuous dose scheme. Thus, they can be recommended when a discrete dose scheme needs to be implemented. However, the original implementation of the CRM design [19] requires initial guesses of the toxicity probabilities for the pre-specified dose set, which could be a daunting task as pointed out by Lee and Cheung [37] even if 9 to 11 doses are used.

The continuous dose scheme showed equal or better results than the discrete dose schemes D0.05, D0.10, D0.125, D0.25 for samples sizes of 20, 40, and 60 patients. The only exception is the dose scheme D0.20, which presented the best results for optimal MTD and toxicity criteria surpassing all dose schemes. Such performance is not a surprise, considering that D0.20 contains the smallest set of pre-specified doses such that each dose is the true MTD for at least 4 out of 16 possible scenarios. Furthermore, caution should be taken when continuous dose is used in the CRM design because an excessive median value for the average DLT was observed.

Recent works [17, 18] presented ideas to add new doses during the trial into the pre-specified discrete dose scheme. Based on the simulations, if there is not enough knowledge about the true MTD value as commonly happens in the real world, continuous dose scheme is advocated as an attractive and more genuine option to discrete dose schemes, which may require protocol amendments to add new doses in the course of the trial.

## Supporting information

S1 File R Code. R Code used for the simulations.
(ZIP)

S2 File Data. Data obtained from the simulations.
(ZIP)
